# An analysis of client complaints and their effects on veterinary support staff

**DOI:** 10.1002/vms3.725

**Published:** 2022-01-19

**Authors:** Charles W. Rogers, Lisa A. Murphy, Ruth A. Murphy, Kylee A. Malouf, Rachel E. Natsume, Briana D. Ward, Colleen Tansey, Reid K. Nakamura

**Affiliations:** ^1^ MedVet McMurray Pennsylvania USA; ^2^ Friendship Hospital for Animals Washington District of Colombia USA; ^3^ Beamount Hospital Dublin Ireland; ^4^ Midwestern University College of Pharmacy Downers Grove Illinois USA; ^5^ University of Sydney School of Veterinary Medicine Camden New South Wales Australia; ^6^ Philadelphia Osteopathic School of Medicine Philadelphia Pennsylvania USA; ^7^ VCA West Los Angeles Animal Hospital Los Angeles California USA; ^8^ Idexx Laboratories Westbrook Maine USA

**Keywords:** burnout, clinical epidemiology, complaints, education, statistics, support staff

## Abstract

**Background:**

Veterinarians and support staff have been reporting the negative mental health effects from client complaints (CC). A previous study was performed evaluating these effects in veterinarians however no such study has been performed on veterinary support staff (VSS).

**Objectiv:**

The purpose of this study was to investigate the frequency and effects of CCs on VSS.

**Methods:**

A cross sectional anonymous survey using a web‐based questionnaire was created evaluating the frequency, type, and effects from CC. The survey was distributed to five different VSS Facebook groups and respondents provided demographic information and reported the frequency and effects of such complaints.

**Results:**

A total of 681 questionnaires were collected during the study period but 130 were incomplete and were excluded from analysis. This resulted in 551 completed questionnaires available for review. One hundred and ninety (34.4%) VSS reported being subject to a CC in the previous 6 months with cost of care the most common reason (78.6%). Two hundred and sixty VSS (47.2%) reported feeling depressed because of CCs made against them, 295 VSS (53.5%) stated CCs negatively affected their enjoyment of their job, and 146 (26.5%) have considered changing their career because of CCs.

**Conclusions:**

CC have detrimental effects on VSS career satisfaction, mental health and hospital practices. Further studies are warranted to mitigate the detrimental effects of CCs.

## Graphical abstract

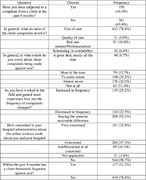

## 1 INTRODUCTION

With the recent global pandemic in particular, several studies have shown the high stress health care workers are experiencing (Huo et al., 2021; Matsuo et al., 2020; Yamada et al., 2021). Working in both human and veterinary health care exposes workers to excessive stress due to the constant direct contact with sometimes critically ill patients and their families. Professional burnout is a syndrome that develops against a background of such chronic stressors leading to the depletion of the emotional energy of the affected working person (Sweileh, 2020). Characteristics of burnout include the following: the feeling of emotional exhaustion where the person cannot give themselves to work as before, dehumanisation where they develop a negative attitude towards patients and a lack of professionalism (Aronsson et al., 2017; Chemali et al., 2019; Sweileh 2020). Burnout was common among veterinary technicians in specialty teaching hospitals and was positively associated with perceived medical errors, desire to change careers and depression (Hayes et al., 2020). Job stress is identified as the principal cause of burnout and job stressors for veterinary technicians include interpersonal relationships, workload and work scheduling (Foster & Mapes, 2014; Hayes et al., 2020; Leiter & Durup, 1994). A previously unexamined stressor is client complaints (CCs). A recent study evaluated the frequency and effects of CCs among small animal veterinary internists (Bryce et al., 2019). While CCs were relatively rare, the effect of CCs was pronounced, with more than 70% of small animal veterinary internists reporting feeling depressed due to a CC, and more than 40% considering changing their career because of CCs. Such complaints have been more thoroughly evaluated in human medicine among the many medical specialities. A commonly cited cause of complaints and even litigation is poor patient outcomes combined with breakdowns in communication between physicians and their patients and families (Levinson et al., 1997). Studies in human medicine showed dissatisfied patients cited feeling rushed, ignored, receiving inadequate explanations and spending less time with patients during routine visits as common causes of breakdown in communication (Hickson et al., 1994). Veterinary medicine is unique in that both veterinarians and support staff have to also deal with the financial aspect of health care for client's pets (Coe & Bonnett, 2007). There is limited scientific data regarding whether CCs have the same detrimental effects on veterinary support staff (VSS). Given the recent increase in veterinary practice caseload and demand for both veterinarians and support staff could increase the rate of CC, it is warranted to evaluate the effect of such complaints on VSS. The purpose of this study was to evaluate the frequency, nature and consequences of CCs on VSS.

## 2 MATERIALS AND METHODS

The questionnaire (Appendix 1) developed for this study was modified from a previous study (Bryce et al., 2019). A CC was defined as a complaint made by a client directly to the VSS member, a supervisor (i.e., hospital management or attending veterinarian) or online (i.e., Yelp or Facebook). For this study a VSS was defined as a veterinary technician, veterinary assistant, veterinary receptionist or kennel staff member. Demographic information including location, age, gender, clinical experience and type of practice were reported. Additionally, VSS perceptions of CCs, as well as the effects of CCs on the VSS's job satisfaction, mental health and potential for job change was also evaluated. All VSS consented to participating in the study before completing the questionnaire. The study was self‐funded and no external funding was obtained.

The questionnaire was open to members of severe Facebook discussion groups which were primarily aimed at VSS over a 2 month period in 2019. These groups included ‘tech support’, ‘veterinary technicians in small animal clinical practice’, ‘ER vet tech rounds’, ‘veterinary ECC small talk’ and ‘veterinary anaesthesia nerds’. Approval from the administration personnel of each Facebook group was sought prior to posting the questionnaire as to ensure it aligned with the groups’ rules and objectives. The participant's consent was asked prior to starting the questionnaire and they were instructed to participate only once in the study and were free to decline participation. Every 2 weeks, a new post was created to try and increase participant numbers. The questionnaire was open to all respondents regardless of international geographic location.

### 2.1 Questionnaire validation

Face validity, which is the degree to which the questionnaire appears to assess the desired qualities, was established through discussions with veterinarians through feedback after they had completed the questionnaire previously. This is in line with studies evaluating work stress where similar pilot groups of cohorts were asked to assess the questionnaire prior to the study group (Bryce et al., 2019; Frantz et al., 2019; Holmgren et al., 2009; Mucci et al., 2015). After the initial questionnaire was generated, 10 VSS and 10 veterinarians of various clinical experience and specialties completed the questionnaire and expressed they did not have any trouble understanding the survey questions and would not hesitate to complete the survey again if needed. The estimated time to complete the questionnaire through this initial evaluation was approximately 10 min. The study protocol was approved by the investigators’ Hospital's Institutional Review Board and Committee.

### 2.2 Statistical analysis

Responses to survey questions were tabulated and descriptive statistics (number and percentage) were generated.

## 3 RESULTS

Responses were received from 681 VSS during the study period. A survey was considered incomplete if the respondent did not complete the entire survey. One hundred and thirty surveys were incomplete and excluded from analysis. Therefore 551 questionnaires were evaluated. The data were then divided into three different categories: Respondent Demographics, Frequency and Type of CCs and Perception of CCs.

### 3.1 Respondent demographics

The majority of VSS were female (533/551 respondents, 96.7%) veterinary technicians (445/551 respondents, 80.8%) who were less than 40 years of age (387/551 respondents, 70.2%) and had been working in the veterinary field for less than 20 years (450/551 respondents, 81.7%) (see Table 1). Most VSS worked in small animal practice outside of academia (538/551 respondents, 97.6%). The majority of respondents were in the United States and within this region, there was relatively equal distribution between across this country. A minority (90/551 respondents, 16.3%) were international which was defined as outside the United States.

**TABLE 1:** Demographics of respondents (551 total respondents)

Question	Choices	Frequency
Age?	<30 years of age	193 (35%)
	30–39 years of age	194 (35.2%)
	40–49 years of age	105 (19%)
	50–59 years of age	59 (10.7%)
Gender?	Female	533 (96.7%)
	Male	18 (3.3%)
Length working in the veterinary field?	1–9 years	261 (47.3%)
	10–19 years	189 (34.3%)
	20–29 years	78 14.2%)
	>30 years	23 (4.2%)
Primary role at your hospital?	Veterinary Technician	445 (80.8%)
	Veterinary Assistant	80 (14.5%)
	Receptionist	24 (4.3%)
	Kennel Staff	2 (0.4%)
Primary practice type?	Small Animal	538 (97.6%)
	Large Animal	4 (0.8%)
	Academia	9 (1.6%)
Geographic location where you practice?	Northeast USA	109 (19.8%)
	South USA	132 (24.0%)
	Midwest USA	95 (17.2%)
	West USA	125 (22.7%)
	International	90 (16.3%)

### 3.2 Frequency and type of CCs

One hundred and ninety VSS (34.4%) reported receiving a CC in the previous 6 months (see Table 2). The CCs were equally reported to the VSS member directly, veterinarian the individual worked with, the hospital manager or online. Cost of care was by far the most common reason for a CC (433/551 responses, 78.6%). For VSS that received a CC, the majority reported receiving a CC every few months (281/551 responses, 50.9%), however 88.6% (488/551 responses) worried to varying degrees about a CC.

**TABLE 2:** Frequency and type of client complaints (551 total respondents)

Question	Choices	Frequency
Have you been subjected to a complaint from a client in the past 6 months?	Yes	190 (34.4%)
	No	361 (65.6%)
How was the most recent CC Made?	Directly to you	168 (30.4%)
	To the veterinarian you work with	108 (19.6%)
	To your hospital manager/administration	147 (26.7%)
	Online (i.e., YELP)	128 (23.3%)
In general, what do most of the client complaints involve?	Cost of care	433 (78.6%)
	Quality of care	31 (5.0%)
	Bed side manner/Professionalism	51 (10.0%)
	Scheduling (i.e., availability)	36 (6.4%)
How often do clients make a complaint against you (e.g., to you directly, to management, to the veterinarian you work with or online)	Never	170 (30.8%)
	Rarely (once every few months)	281 (50.9%)
	Somewhat frequently (once a month)	34 (6.2%)
	Frequently (once a week)	65 (12.1%)
In general, to what extent do you worry about client complaints being made against you?	A great deal, nearly all the time	48 (8.7%)
	Most of the time	70 (12.7%)
	To some extent	194 (35.2%)
	Almost never	176 (32.0%)
	Not at all	63 (11.4%)
As you have worked in the field and gained more experience how has the frequency of complaints changed?	Increased in frequency	139 (25.2%)
	Decreased in frequency	124 (22.5%)
	Staying the same/no noticeable difference	288 (52.3%)
With regard to online complaints (i.e., Yelp, Facebook) does your hospital attempt to address complaints made by clients (i.e., contact client directly when a complaint appears)?	Yes	374 (67.9%)
	No	67 (12.1%)
	Don't know or not applicable	110 (20.0%)
How concerned is your hospital administration about the online reviews made about you and/your hospital	Very concerned	181 (32.8%)
	Concerned	260 (47.3%)
	Indifferent/not at all concerned	89 (16.1%)
	Not applicable	21 (3.8%)
Do you feel the online reviews made by clients are a fair representation of the service your hospital provides	Yes	111 (20.2%)
	No	392 (71.1%)
	Don't know/not sure	48 (8.7%)
Within the past 6 months has a client verbally assaulted you (e.g., yelled, screamed swore at you, etc?)	Yes	298 (54.1%)
	No	253 (45.9%)
Within the past 6 months has a client physically assaulted you (e.g., grabbed, punched, pushed you at the practice, etc.)	Yes	7 (1.3%)
	No	544 (98.7%)
Within the past 6 months has a client threatened litigation against you?	Yes	117 (21.2%)
	No	434 (78.8%)

Three hundred and seventy‐four VSS (67.9%) stated their hospital attempted to address complaints made online and 441 VSS (80.0%) described their hospital administration as concerned about online reviews. One hundred and eleven VSS (20.2%) felt online reviews were a fair representation of the service they provided.

More than half of the study population (298/551 responses, 54.1%) reported being verbally assaulted by a client in the previous 6 months and a few (7 responses, 1.3%) reported being physically assaulted by a client in the previous 6 months. More than one fifth of respondents (117/551 responses, 21.2%) reported being threatened with litigation by a client in the previous 6 months.

### 3.3 Perception of CCs

Most VSS either strongly agreed (77/551 responses 14%) or agreed (229/551 responses, 41.6%) they felt the need to please clients to avoid a CC (see Table 3). One hundred and sixty‐eight VSS (30.5%) agreed that the only individuals who file an online review complain about the service they receive and 238 VSS (43.2%) felt there is no recourse to address or correct an unfair or malicious review/complaint. In terms of the negative consequences of CCs, 260 VSS (47.2%) reported feeling depressed because of a CC made against them, 295 VSS (53.5%) reported a complaint negatively affecting enjoyment of their job and 146 VSS (26.5%) considered changing their career because of CCs made against them.

**TABLE 3:** Perception of client complaints (551 total respondents). To what extent do you agree with the following statements?

Statement	Strongly Agree	Agree	Neutral	Disagree	Strongly disagree
A veterinarian who receives more complaints than others usually does so because of poor clinical performance	17 (3.0%)	138 (25.0%)	130 (23.6%)	227 (41.2%)	39 (7.1%)
I feel the need to please clients to avoid complaints against me	77 (14.0%)	229 (41.6%)	106 (19.20%)	120 (21.8%)	19 (3.4%)
Receiving a complaint would adversely affect my future prospects/ability to be promoted within the hospital/find a different job	27 (5.0%)	138 (25.0%)	98 (17.7%)	228 (41.4%)	60 (10.9%)
The only people who file an online review complain about the service they receive	19 (3.4%)	149 (27.1%)	102 (18.5%)	235 (42.7%)	46 (8.3%)
There is no recourse to address/correct an unfair/malicious review/complaint	76 (13.8%)	162 (29.4%)	101 (18.3%)	190 (34.5%)	22 (4.0%)
I feel depressed because of the complaints made against me	59 (10.7%)	201 (36.5%)	116 (21.0%)	117 (21.3%)	58 (10.5%)
Complaints made against me have negatively affected my enjoyment of my job	90 (16.3%)	205 (37.2%)	95 (17.2%)	110 (20.0%)	51 (9.3%)
I have considered changing my career because of complaints made against me	59 (10.7%)	87 (15.8%)	79 (14.3%)	184 (33.4%)	142 (25.8%)
Complaints are an opportunity to objectively evaluate our performance from the client's perspective and improve care	39 (7.1%)	284 (51.5%)	136 (24.7%)	79 (14.3%)	13 (2.4%)
I have seen improvements in our hospital management to better address a client complaint	37 (6.7%)	190 (34.5%)	140 (25.4%)	130 (23.6%)	54 (9.8%)
I have seen improvement in veterinary medical practices in response to client complaints	16 (2.9%)	186 (33.8%)	176 (31.9%)	141 (25.6%)	32 (5.8%)
Hospital management is too slow to fire difficult clients	245 (44.5%)	184 (33.4%)	52 (9.4%)	54 (9.8%)	16 (2.9%)
Agreeing to clients’ complaints encourages future complaints	143 (25.95%)	218 (39.56%)	117 (21.23%)	72 (13.06%)	1 (0.18%)
Veterinarians should be taught conflict resolution to better handle difficult clients	233 (42.3%)	265 (48.1%)	36 (6.5%)	15 (2.7%)	2 (0.4%)
Veterinary Support staff should be taught conflict resolution to better handle difficult clients	250 (45.4%)	265 (48.1%)	26 (4.7%)	8 (1.4%)	2 (0.4%)

In contrast to the detrimental effects of CCs, more than half of VSS (323/551 responses, 58.6%) felt that CCs were an opportunity to objectively evaluate performance from the client's perspective and 227 VSS (41.2%) reported seeing improvements in hospital management to better address CCs. Four hundred and ninety‐eight VSS (90.3%) either strongly agreed or agreed that veterinarians should be taught conflict resolution to better manage difficult clients and 515 VSS (93.5%) either strongly agreed or agreed that VSS should be taught conflict resolution to better manage difficult clients.

## 4 DISCUSSION

CCs had a profound detrimental effect on VSS mental health and career satisfaction as 47.2% reported feeling depressed because of a CC made against them, 53.5% reported a complaint negatively affecting enjoyment of their job, and 26.5% considered changing their career because of CCs made against them. Although approximately one‐third of VSS (34.4%) reported a CC in the previous 6 months, 88.6% worry about CC to some extent with 118 (21.4%) describing their worry as ‘most of the time’ or ‘all the time’. As such while CC may be relatively infrequent, the detrimental effects are pronounced and can be a source of chronic anxiety and stress for VSS. In addition, more than half of VSS reported being verbally assaulted by a client in the previous 6 months, more than one‐fifth of VSS reported being threatened with litigation by a client and there was even a small percentage that reported being physical assaulted by clients. This may explain why 429 (77.9%) feel hospital management is too slow to fire difficult clients. Better attention and support for all members of the veterinary community is critical to minimise the negative impact of CCs and the resultant stress and depression.

The majority of VSS were female and less than 40 years of age, similar to a previous study evaluating burnout in veterinary specialty teaching hospitals (Hayes et al., 2020). Previous studies in health care workers have found females are more empathic and report higher levels of stress compared to men (Antoniou et al., 2003). Women may also be more focused on their emotions and rely on the social support of their peers to deal with such stress (Gabriel et al., 2016; Lim et al., 2010). Studies in human medicine report younger individuals may also be more affected by CCs (Cheung & Yip, 2015; Xie et al., 2011). As such, VSS may have a higher susceptibility to the negative effects of CCs requiring further management and support by veterinarians and hospital administration to alleviate such stressors. One potential avenue is to include conflict resolution which may be a means to better mitigate client concerns without progressing to an actual CC. Indeed, in this study 90.4% of VSS agreed that veterinarians and 93.5% felt VSS should be taught conflict resolution techniques to better handle difficult clients. Additional studies to better manage clients and mitigate the job stressors in veterinary practice appear warranted.

Almost 50% of respondents in this study agreed or strongly agreed that they feel depressed because of CCs made against them. This was a self‐reported diagnosis, and the authors did not attempt to objectively score depression levels in VSS as has been previously reported (Hayes et al., 2020). The authors also did not attempt to evaluate prevalence of burnout in this population although a positive association between burnout and depression has been previously reported suggesting burnout could also be common in this study population as well (Bianchi et al., 2015; Hayes et al., 2020).

Previous studies in human medicine have reported that CCs have the potential to improve medical practices (Gillepsie & Reader, 2016; Harrison et al., 2016). These findings were echoed in the present study with (58.6%) respondents who agreed that CCs are an objective means to evaluate performance. Indeed, respondents reported witnessing improvements in both hospital management (227 VSS, 41.2%) and medical practices (202 VSS, 36.7%) in response to CCs. The specific type of improvements was not evaluated in this study, but this finding suggests that there are some beneficial effects to receiving and improving practices. Such a positive reaction to CC likely instils the ability for the personnel to learn from their mistakes and may reduce underreporting of negligence or malpractice. Analysing and responding to CCs has been demonstrated in human medicine to improve the quality of health care (Gillepsie & Reader, 2016; Harrison et al., 2016). The potential benefit of CCs to improve medical practices in veterinary hospitals requires further evaluation.

There are several limitations to the study reported here. The investigators did not have access to the membership rosters of the different Facebook groups and the results were anonymous so we cannot be sure all who answered were VSS. Another limitation was the predominantly female responses over male which could have affected the results as previously described in this discussion. VSS may be reluctant to disclose a CC due to professional or personal embarrassment and thus the frequency of CCs among VSS may be higher than is reported here. The investigators attempted to encourage forthright answers by guaranteeing anonymity although there is no means to verify the self‐reported numbers here. Two of the Facebook groups used to enrol VSS focused specifically on emergency and critical care technicians which may have biased the results towards a more stressful and strenuous work environment; it would have been more ideal to enrol technicians from a broad category of specialties and practice environments to provide a better cross section among all practice backgrounds. The large majority of the respondents were from small animal hospitals outside academia, and it would have been interesting to compare responses to large animal facilities and/or academic environments. In addition, it would have been interesting to compare geographical differences or comparing VSS of different clinical experience as it is difficult to know if certain locations have higher litigation rates for CC against veterinarians. There is also the ‘healthy worker effect’ which suggests individuals who are unwell tend to leave the job resulting in an overall ‘healthy’ bias in the remaining population (Li & Sung, 1999).

## 5 CONCLUSION

In conclusion, CCs have detrimental effects on career satisfaction, mental health and perceived medical practices among VSS. Given the adverse health effects of burnout on both clients and providers alike as well as the stressors from the recent global pandemic and its effects on veterinary medicine, more attention needs to be paid to veterinarian and VSS well‐being. It is important for VSS to recognise while they work to fulfil their professional responsibilities, they should also recognise the importance of how workplace stressors may affect their mental and emotional well‐being. Further investigations into the effect of CC on other VSS populations as well as better efforts to assess burnout more consistently are warranted which will likely improve career satisfaction and working environment. Programs should be designed aimed at raising awareness, promoting well‐being and burnout prevention, and improving coping with burnout symptoms. Finally, programs centred on conflict resolution should also be included early in the careers of both support staff and veterinarians so they can have better footing dealing with difficult work environments.

## AUTHOR CONTRIBUTIONS

Charles Rogers: conceptulalisation, formal analysis, investigation, methodology; Lisa Murphy: conceptulalisation, formal analysis, investigation, methodology, writing original draft and revisions; Ruth Murphy: formal analysis and consultation; Kylee Malouf: formal analysis, investigation; Rachel Natasume: formal analysis, investigation; Briana Ward: formal analysis, investigation; Coleen Taney: formal analysis, investigation, methodology; Reid Nakamura: conceptulalisation, formal analysis, investigation, methodology, writing original draft and revisions.

## CONFLICT OF INTEREST

There are no potential conflicts of interest

## ETHICAL STATEMENT

The study protocol was approved by the investigators’ Hospital's Institutional Review Board and Committee.

### PEER REVIEW

The peer review history for this article is available at https://publons.com/publon/10.1002/vms3.725

## Data Availability

Data is available with request.

## APPENDIX 1

### 1.1

This study is intended to investigate client complaints and their effects on veterinary support staff and how it affects their quality of life. A recent study showed severe detrimental effects of client complaints on veterinary internist's quality of life and medical practices. We would like to evaluate these effects on veterinary support staff. IF YOU DO NOT REGUARLY INTERACT WITH CLIENTS (IE RESEARCH SETTING) PLEASE DO NOT PARTICIPATE IN THIS SURVEY.

All information that you provide is ANONYMOUS and CONFIDENTIAL and held in the strictest confidence. You will not be asked to provide any information that can be used to identify you nor can you be identified by the investigators by filling in any part of this survey. As such please answer each question honestly!

This is an electronic form of consent for the study. By ticking the boxes below, you agree to participate in the study.

1. I consent to the use of my survey results to better understand the impact of client complaints on veterinary support staff and their quality of life.
  - Yes
  - No
2. What is your age
  - < 30 years of age
  - 30‐39 years of age
  - 40‐49 years of age
  - 50‐59 years of age
  - 60+ years of age
3. What is your gender
  - Female
  - Male
4. How many years have you been involved in the veterinary field?
  - 1‐9 years
  - 10‐19 years
  - 20‐29 years
  - 30 years
5. What best describes your primary role at your hospital?
  - Receptionist
  - Veterinary Assistant
  - Veterinary Technician
  - Kennel Staff
6. What best describes your primary practice type?
  - Small animal
  - Large Animal
  - Academia
7. What best describes your geographic location where you practice?
  - West (Montana, Wyoming, Colorado, New Mexico, Idaho, Utah, Arizona, Nevada, Washington, Oregon, California, Alaska, Hawaii)
  - Mid‐West (Illinois, Indiana, Iowa, Kansas, Michigan, Minnesota, Missouri, Nebraska, North Dakota, Ohio, South Dakota, Wisconsin)
  - Northeast (Maine, New York, New jersey, Rhode Island, Pennsylvania, New Hampshire, Conneticut, Vermont, Massachusetts
  - South (Florida, Georgia, North Carolina, South Carolina, Virginia, West Virginia, Maryland, Delaware, Alabama, Kentucky, Arkansas, Louisiana, Oklahoma, Texas, Mississippi, Tennessee)
  - Other (ie outside the US)
8. Have you been subjected to a complaint from a client in the past 6 months
9. Yes
10. No
11. Regarding your most recent complaint, how was it made
12. Directly to you
13. To the veterinarian you work with
14. To your hospital manager/administration
15. Online (ie Yelp)
16. In general what do most of the client complaints involve
17. Cost of care
18. Quality of care
19. Bed Side manner/Professionalism
20. Scheduling (ie availability)
21. How often do clients make a complaint against you (ie to you directly, to management, to the veterinarian you work with or online)
22. Never
23. Rarely (once every few months)
24. Somewhat frequently (once a month)
25. Frequently (once a week)
26. In General, to what extent do you worry about client complaints being made against you?
27. A great deal, nearly all the time
28. Most of the time
29. To some extent
30. Almost never
31. Not at all
32. As you have worked in the field and gained more experience how has the frequency of complaints changed?
33. Increased in frequency
34. Decreased in frequency
35. Staying the same/no noticeable difference
36. With regard to online complaints (ie Yelp, Facebook) does your hospital attempt to address complaints made by clients? (ie contact client directly when a complaint appears)
37. Yes
38. No
39. Don't know or Not applicable
40. How concerned is your hospital administration about the online reviews made about you and/or your hospital
41. Very concerned
42. Concerned
43. Indifferent/not at all concerned
44. Not applicable
45. Do you feel the online reviews made by clients are a fair representation of the service your hospital provides?
46. Yes
47. No
48. Don't know/not sure
49. Within the past 6 months has a client verbally assaulted you (ie yelled, screamed, swore at you, etc)?
50. Yes
51. No
52. Within the past 6 months has a client physically assaulted you (ie grabbed, punched, pushed you at the practice, etc)?
53. Yes
54. No
55. Within the past 6 months has a client threatened litigation against you?
56. Yes
57. No
58. To what extent do you agree with the following statements

**Table:**

Statement	Strongly Agree	Agree	Neutral	Disagree	Strongly Disagree
A veterinarian who receives more complaints than others usually does so because of poor clinical performance					
I feel the need to please clients to avoid complaints against me					
Receiving a complaint would adversely affect my future prospects/ability to be promoted within the hospital/find a different job					
The only people who file an online review complain about the service they receive					
There is no recourse to address/correct an unfair/malicious review/complaint					
I feel depressed because of the complaints made against me					
Complaints made against me have negatively affected my enjoyment of my job					
I have considered changing my career because complaints made against me					
Complaints are an opportunity to objectively evaluate our performance from the client's perspective and improve care					
I have seen improvements in our hospital management to better address a client complaints					
I have seen improvement in veterinary medical practices in response to client complaints					
Hospital management is too slow to fire difficult clients					
Agreeing to s client's complaints encourages future complaints					
Veterinarians should be taught conflict resolution to better handle difficult clients					
Veterinary support staff should be taught conflict resolution to better handle difficult clients					

21) Within the LAST 6 MONTHS, have you ever seen a veterinarian you work with do any of the following that you felt was to avoid a client complaint and it's effects ie disciplinary actions by managers, being sued, or negative publicity in the media/internet?

**Table:**

Statement	All the time	Often	Sometimes	Rarely	Never
Change the way they practice medicine					
Prescribed more medications than medically indicated					
Consented to treatment requested/demanded by the owner even though they did not feel it was necessary					
Offered invasive procedures against professional judgement at the owner's request/demand					
Admitted an animal to the hospital when the animal could have been discharged home safely or managed as an outpatient					
Carried out more tests than necessary					
Avoided a particular type of procedure					
Not accepted “high risk” patients in order to avoid possible complications					
Discounted services (ie tests, medications) for a client to avoid a complaint even if they thought the complaint had no merit					
Provided free services (ie tests, medications) to avoid or resolve a complaint with a client even if they thought the complaint had no merit					
